# Laparotomic radical hysterectomy versus minimally invasive radical hysterectomy using vaginal colpotomy for the management of stage IB1 to IIA2 cervical cancer: Survival outcomes

**DOI:** 10.1097/MD.0000000000028911

**Published:** 2022-02-25

**Authors:** Eun Jung Yang, Nae Ry Kim, A. Jin Lee, Seung-Hyuk Shim, Sun Joo Lee

**Affiliations:** Department of Obstetrics and Gynaecology, Konkuk University Hospital, Seoul, Korea.

**Keywords:** cervical cancer, laparotomy, minimally invasive surgery, oncological outcomes, radical hysterectomy

## Abstract

This study compared survival outcomes for patients with stage IB1 to IIA2 (International Federation of Gynecology and Obstetrics stage 2009) cervical cancer who underwent open radical hysterectomy (ORH) versus those who underwent minimally invasive radical hysterectomy (MIRH) using vaginal colpotomy (VC).

Data for 550 patients who were diagnosed with cervical cancer at our institution during the period August 2005 to September 2018 was retrospectively reviewed. Of these, 116 patients who underwent radical hysterectomy (RH) were selected after applying the exclusion criteria. All MIRH patients underwent VC. Clinicopathological characteristics and survival outcomes between the ORH and MIRH groups were compared using appropriate statistical testing.

Ninety one patients were treated with ORH and 25 with MIRH during the study period. Among the MIRH patients, 18 underwent laparoscopy-assisted radical vaginal hysterectomy and 7 underwent laparoscopic RH. Preoperative conization was performed more frequently in MIRH patients than in ORH patients (44% vs 22%, respectively, *P* = .028). The incidence of lymph node invasion was higher in the ORH group than in MIRH group (37.4% vs 12.0% respectively; *P* = .016). Following RH, ORH patients underwent adjuvant treatment more frequently than MIRH patients (71.4% vs 56.0%, respectively, *P* = .002). There were no significant differences between ORH and MIRH patients for either progression-free survival (PFS) (91.3% vs 78.7%, respectively; *P* = .220) or 5-year overall survival (OS) (96.6% vs 94.7%, respectively, *P* = .929). In univariate analysis, lympho-vascular space invasion was the only clinicopathological feature associated with decreased PFS. No other clinicopathological factors was significantly associated with PFS or OS in univariate and multivariate analyses.

Despite a higher incidence of unfavorable prognostic factors in ORH patients, their survival outcomes were not different to those of MIRH patients with VC.

## 1 Introduction

Cervical cancer is the second most common female cancer worldwide and the sixth most common female cancer in Korea.^[1–3]^ It is the third highest cause of female cancer deaths worldwide and the seventh highest cause of female cancer deaths in Korea.^[1–3]^

Radical hysterectomy (RH) with pelvic lymph node dissection is the surgical treatment of choice for cervical cancer patients diagnosed with stage IB1 to IIA2 of the disease as classified by the International Federation of Gynecology and Obstetrics (FIGO) in 2009.^[4,5]^ Adjuvant radiotherapy or concurrent chemoradiation therapy (CCRT) is also recommended to these patients depending on the risk factors.^[6–9]^

RH may be performed with open radical abdominal hysterectomy (ORH) or with minimally invasive radical hysterectomy (MIRH). The MIRH procedure is performed by laparoscopy as well as robot and appears to be oncologically safe in existing clinical practice.^[10–12]^ However, the results of a prospective randomized trial published by Ramirez et al showed that MIRH was associated with shorter disease-free and overall survival compared to ORH.^[13]^ These authors suggested that possible reasons for the worse outcome in MIRH patients were the use of uterine manipulation, the effect of insufflation gas (CO_2_), and intracorporeal colpotomy. Since the original report, several retrospective studies also found superior oncologic outcomes for ORH.^[15–17]^ In 2020, the cervical cancer surgery, observation, retrospective (SUCCOR) study reported that MIRH was associated with an increased risk of recurrence and death compared to ORH.^[14]^ In contrast, several other studies have reported that oncologic outcomes for MIRH were not inferior to ORH.^[18–21]^ In 2020, the European Society of Gynaecological Oncology stated that open surgery should be the standard treatment, and that patients must be informed about the available evidence on survival, complications, and quality of life relating to the 2 surgical approaches. With regard to MIRH every effort should be made to avoid the spillage of tumor cells into the peritoneal cavity.^[22]^

The present retrospective study was performed in order to compare the oncologic outcomes between cervical cancer patients who underwent ORH or MIRH. Vaginal colpotomy and cuff closure, uterine manipulation and CO_2_ insufflation were performed for all MIRH cases. We believe this study represents real-world practice and adds more evidence to our knowledge of vaginal colpotomy in MIRH.

## 2 Methods and materials

This study was approved by the Institutional Review Board (IRB) of Konkuk University hospital (KUMC 2020-07-050). The requirement for written informed consent was waived by the IRB.

### 2.1 Study population

**Figure 1:**
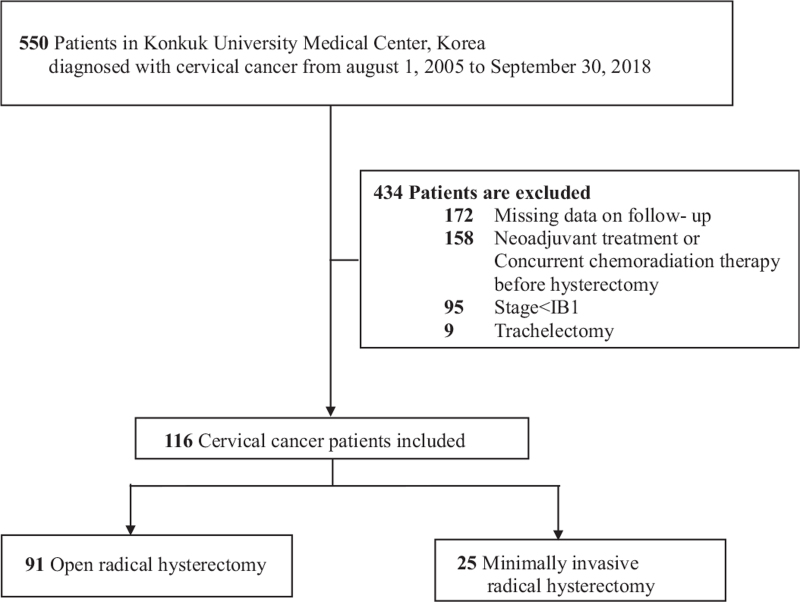
Flowchart of study population.

Cervical cancer patients who underwent primary treatment at Konkuk University Hospital between August 2005 and September 2018 were retrospectively reviewed. All cases were classified according to the 2009 FIGO cervical cancer staging criteria. Of the total 550 patients diagnosed with cervical cancer, those meeting the following criteria were included (Fig. 1):

1. diagnosed with FIGO stage IB1-IIA2; and
2. underwent primary RH.

The exclusion criteria were:

1. insufficient clinical and/or pathologic data;
2. received neoadjuvant treatment or CCRT prior to RH; or
3. received radical trachelectomy or cervical cancer was detected after simple hysterectomy.

All eligible patients who underwent RH since the opening of our institute in August 2005 were enrolled. Patients who met the study inclusion criteria were divided into 2 groups: those who underwent RH by conventional laparotomic surgery (ORH group), and those who underwent RH by laparoscopic surgery (MIRH group).

### 2.2 Surgical methods

Surgery was performed by suitably trained surgeons from our institution. The surgical methods were determined according to the patient's needs following explanation of the preoperative examination results and the strong and weak points of each surgical approach. Vaginal colpotomy was performed in our institute during laparoscopic-assisted radical vaginal hysterectomy (LARVH).^[23]^ or laparoscopic radical hysterectomy (LRH). LRH was based on the same procedure as described in previous studies,^[24–26]^ except for the vaginal colpotomy and cuff closure. In LRH, colpotomy was performed by accessing the vagina after completing the intracorporeal procedures. Following removal of the uterus, the peritoneum and vaginal opening were closed by accessing the vagina. All patients in the ORH group underwent Piver-Rutledge type 3 hysterectomy and pelvic lymph node dissection.^[5]^ Gynecologic oncology surgeons in our institute performed both MIRH and ORH. Following RH, adjuvant CCRT was recommended when one or more pathologic risk factors was present (i.e., the involvement of lymph nodes, parametrium, or resection margin).^[8]^ Adjuvant radical hysterectomy was recommended for node-negative, margin-negative, parametrium-negative cases according to Sedlis criteria (i.e., the presence of 2 or more intermediate risk factors: lympho-vascular space invasion (LVSI), stromal invasion, and tumor size).^[9]^ Patients diagnosed with cervical cancer in our institute commonly undergo abdominal CT, pelvis MRI and PET-CT. Imaging is usually performed between the first diagnosis (punch biopsy or conization) and RH, and provides additional information on cervical cancer size, risk of parametrial invasion and lymph node involvement. Progression-free survival (PFS) was defined as the elapsed time from the date of diagnosis to the date of relapse or censoring, while overall survival (OS) was defined as the elapsed time from the date of diagnosis to the date of death, last follow-up, or censoring.

### 2.3 Statistical analysis

Frequency distributions were compared using the Chi-Squared and Fisher exact tests. The Kaplan–Meier method was used to estimate PFS and OS. Analysis of the prognostic significance of surgical methods and of clinicopathological factors was performed using the Cox proportional hazards model. The IBM SPSS statistics 22.0 (IBM SPSS Statistics, Chicago, IL) package was used for all statistical analyses, with. *P* < .05 considered statistically significant.

## 3 Results

### 3.1 Clinical data

During the study period, 91 patients underwent ORH and 25 underwent MIRH, with the latter comprising 18 LARVH and seven LRH cases (Fig. 1). The median follow-up period was 60.8 months (range, 0.8–182.7). During the study period, patients with cervical cancer stage IIA2 underwent CCRT and hence none of the enrolled patients had stage IIA2 disease. None of the patients who underwent MIRH was converted to ORH. Table 1 shows the clinicopathological characteristics of all enrolled cases in the ORH and MIRH groups. There were no significant differences between the 2 groups in terms of patient age, histologic subtypes, FIGO stage, parametrial invasion, resection margin involvement, LVSI and depth of invasion. Of the total 116 patients, 87 (75%) were classified as having stage IB1 disease (≤ 4 cm in greatest dimension), comprising 67 ORH patients (67/91, 73.6%) and 20 MIRH patients (20/25, 80.0%). Preoperative conization was performed when a high-grade squamous intraepithelial lesion or cancer was reported from Papanicolaou smear or biopsy without an obviously visible cancer lesion. Thirtyone patients underwent preoperative conization during the study period, comprising 20 ORH patients (20/91, 22.0%) and 11 MIRH patients (11/25, 44.0%). Of these, 26 (83.9%) were identified as being margin positive. MIRH patients thus underwent pre-operative conization more frequently than ORH patients (*P* = .028). Of the 31 patients who underwent conization, 26 (83.9%) had FIGO stage 1B1 disease (17 ORH, 9 MIRH). However, there was no significant difference between stage IB1 and stage IB2-IIA2 patients in terms of the frequency that underwent conization (*P* = .230). A higher incidence of lymph node invasion was observed in the ORH group compared to the MIRH group (37.4% vs 12.0%, respectively, *P* = .016). Following RH, 65 patients (71.4%) in the ORH group and 14 patients (56.0%) in the MIRH group received adjuvant treatment, with the rate of treatment being significantly higher in ORH patients (*P* = .002). (Table 1)

**Table 1:** Baseline characteristics of enrolled patients.

Characteristics	ORH (n = 91)	MIRH (n = 25)	*P* value
Age, yr			
Mean ± SD	49.4 ± 11.3	47.9 ± 10.0	.389
<50, n (%)	54 (59.3)	13 (52.0)	.648
≥50, n (%)	37 (40.7)	12 (48.0)	
Histologic subtype, n (%)			.398
Squamous-cell carcinoma	59 (64.8)	19 (76.0)	
Adenocarcinoma	21 (23.1)	4 (16.0)	
Adenosquamous carcinoma	10 (11.0)	1 (4.0)	
Small cell carcinoma	1 (1.1)	1 (4.0)	
Stage of disease, n (%)			.309
IB1	67 (73.6)	20 (80.0)	
IB2	15 (16.5)	5 (20.0)	
IIA1	9 (9.9)	0	
IIA2	0	0	
Pre-operation conization, n (%)			.028
No	71 (78.0)	14 (56.0)	
Yes	20 (22.0)	11 (44.0)	
Risk factors, n (%)			
Parametrial involvement	16 (17.6)	4 (16.0)	1.000
Resection margin involvement	12 (13.2)	0	.067
Lymph node involvement	34 (37.4)	3 (12.0)	.016
LVSI	42 (46.2)	13 (52.0)	.655
Invasion depth ≥ 1/2	61 (67.0)	14 (56.0)	.349
Adjuvant treatment, n (%)			.002
None	26 (28.6)	11 (44.0)	
Chemotherapy only	5 (5.5)	6 (24.0)	
Radiation only	8 (8.8)	3 (12.0)	
CCRT	52 (57.1)	5 (20.0)	
Recurrence, n (%)	8 (8.8)	5 (20.0)	.150
Died of disease, n (%)	3 (3.3)	1 (4.0)	1.000

CCRT = concurrent chemoradiation therapy, LVSI = lymphovascular space invasion, MIRH = minimally invasive radical hysterectomy, ORH = open radical hysterectomy.

### 3.2 Survival outcomes

Eight patients (8.8%) in the ORH group and 5 patients (20.0%) in the MIRH group developed recurrent disease. During the study period, 3 patients (3.3%) in the ORH group and 1 (4.0%) in the MIRH group died of recurrent disease (Table 1). There was no significance difference in either PFS (*P* = .220) or OS (*P* = .929) between the 2 groups (Fig.2A and B). Patients with stage IB1 disease showed similar survival outcomes regardless of the surgical method used (*P* = .820 and *P* = .560 for PFS and OS, respectively) (Fig. 2 C and D). Patients with FIGO stage IB2 to IIA2 disease who underwent ORH showed a trend for e better PFS compared to MIRH patients (90.5% vs 60.0%), although this did not reach statistical significance (*P* = .107) (Fig. 2 E). There was no difference in OS between MIRH and ORH patients with FIGO stage IB2 to IIA2 disease (93.8% vs 75.0%, *P* = .302) (Fig. 2 F). Histologic subtypes had no significant impact on patient survival (data not shown). Table 2 presents the results of the survival analysis. Univariate analysis showed that LVSI was associated with significantly shorter PFS (HR, 3.789: 95% CI, 1.04–13.80, *P* = .043). None of the other clinicopathological factors were significantly associated with PFS or OS in univariate and multivariate analyses. The site of cancer recurrence was different according to the surgical approach used; however, this was not statistically significant (*P* = 1.000) (Table 3). For patients who underwent MIRH, the site of recurrence was vaginal stump (2 patients), adnexa (1 patient) and distant metastases (2 patients: 1 para-aortic lymph node and 1 lung), while for patients who underwent ORH it was vaginal stump (1 patient), pelvic cavity (3 patients) and distant metastases (4 patients: 2 liver, 1 para-aortic lymph node and one lung).

**Figure 2:**
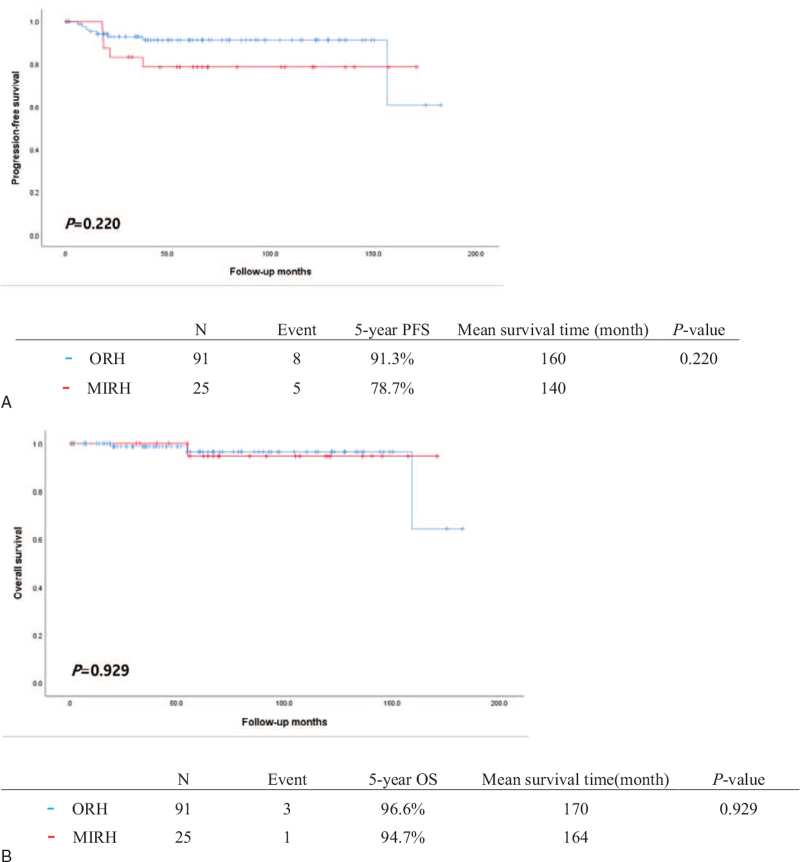
Progression-free survival (PFS) and overall survival (OS) of patients treated by open radical hysterectomy (ORH) or minimally invasive radical hysterectomy (MIRH) in the study population. All patients: (A) PFS, (B) OS. Patients with FIGO stage IB1 (≤4 cm in greatest dimension): (C) PFS, (D) OS. Patients with FIGO stage IB2-IIA2: (E) PFS, (F).

**Figure 2 (Continued):**
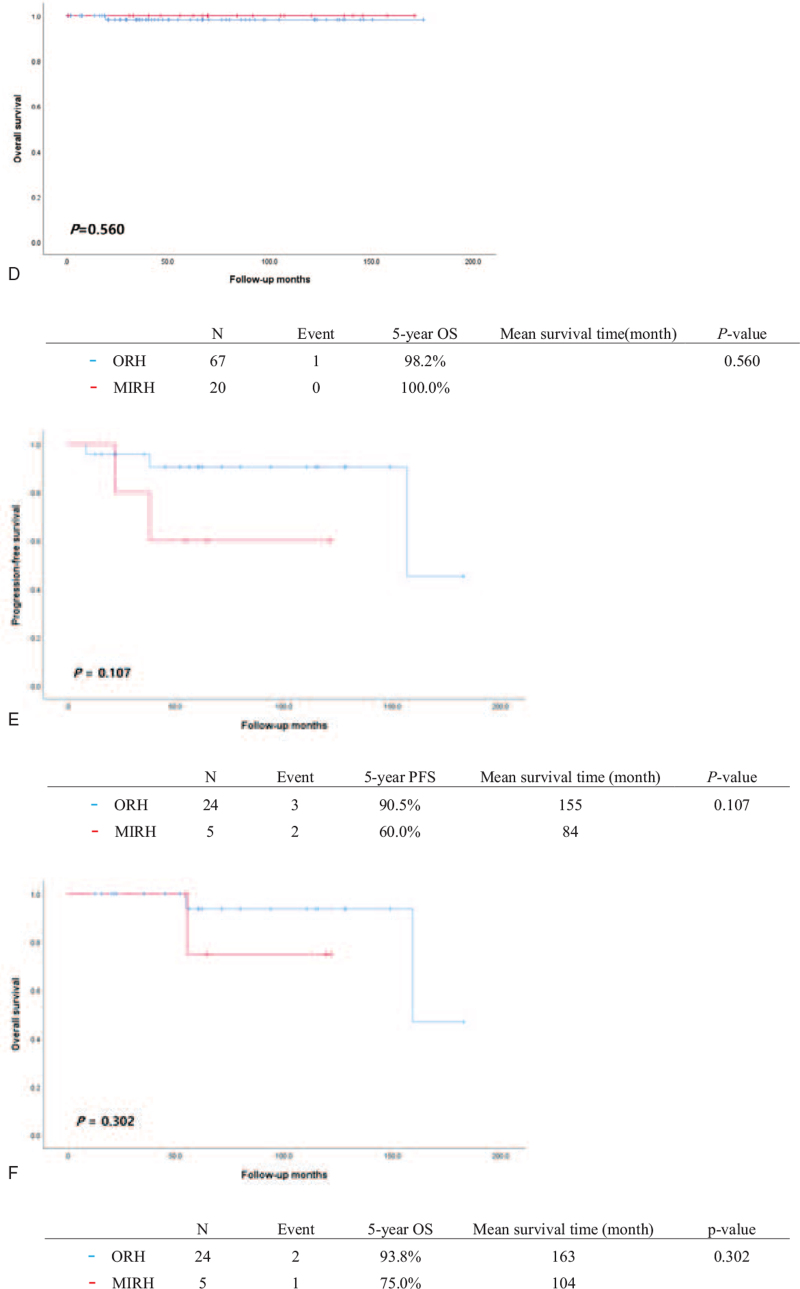
Progression-free survival (PFS) and overall survival (OS) of patients treated by open radical hysterectomy (ORH) or minimally invasive radical hysterectomy (MIRH) in the study population. All patients: (A) PFS, (B) OS. Patients with FIGO stage IB1 (≤4 cm in greatest dimension): (C) PFS, (D) OS. Patients with FIGO stage IB2-IIA2: (E) PFS, (F).

**Table 2:** Factors associated with progression-free survival in patients with FIGO stage IB1-IIA2.

		Univariate analysis			Multivariate analysis		
Factors	N	HR	95% CI	*P*	Adjusted HR	95% CI	*P*
Age							
<50	67						
≥50	49	0.920	0.292–2.898	.886	0.631	0.178–2.241	.476
Histology							
SCC	78						
Non- SCC	38	1.045	0.315–3.471	.943	1.586	0.424–5.938	.493
Cervical mass							
<4 cm	88						
≥4 cm	28	2.290	0.749–7.002	.146	1.594	0.378–6.727	.526
FIGO stage							
IB1	87						
IB2-IIA2	29	0.781	0.446–1.368	.387	1.122	0.587–2.147	.728
Pre-op conization							
No	85						
Yes	31	0.501	0.110–2.287	.372	0.798	0.150–4.231	.791
PM involvement							
No	96						
Yes	20	2.106	0.648–6.848	.216	0.882	0.184–4.222	.875
RM involvement							
No	104						
Yes	12	1.364	0.287–6.486	.697	0.987	0.168–5.788	.989
LN involvement							
No	79						
Yes	37	2.439	0.810–7.345	.113	2.446	0.538–11.115	.247
LVSI							
No	61						
Yes	55	3.789	1.040–13.800	.043	1.876	0.410–8.587	0.418
Invasion depth							
<1/2	41						
≥1/2	75	3.390	0.751–15.298	.112	2.718	0.367–20.099	.327
Adjuvant treatment							
No	37						
Yes	79	4.836	0.624–37.465	.131	1.751	0.145–21.094	.659
Surgical approach							
ORH	91						
MIRH	25	1.055	0.283–3.931	0.240	2.022	0.449–9.112	.359

CI = confidence interval, HR = hazard ratio, LN = lymph node, MIRH = minimally invasive radical hysterectomy, ORH = open radical hysterectomy, PM = parametrium, RM = resection margin, SCC = squamous cell carcinoma.

**Table 3:** Sites of recurrence according to surgical approach.

Sites of recurrence	ORH (n = 8)	MIRH (n = 5)	*P* value
Vaginal stump / pelvic cavity, n (%)	4 (50.0)	3 (60.0)	1.000
^∗^Distant metastases, n (%)	4 (50.0)	2 (20.0)	

MIRH = minimally invasive radical hysterectomy ∗Distant metastases: lymph nodes, liver, lung, bone; ORH = open radical hysterectomy.

## 4 Discussion

The present study indicates that the oncological outcomes for patients with stage IB1 to IIA2 cervical cancer were similar after ORH or MIRH followed by tailored adjuvant therapy. Neither stage IB1 or stage IB2-IIA2 groups showed any significant difference in survival according to the surgical technique used, although there was a trend for stage IB2-IIA2 patients to have worse PFS with MIRH.

Possible reasons for the similar survival outcomes observed between ORH and MIRH for stage IB1-IIA2 patients are discussed below. First, the higher frequency of preoperative conization in the MIRH group could affect oncological outcomes (Table 1). In general, cervical cancer diagnosed by conization can mean smaller volume or earlier stages of disease. Moreover, a previous study by Kim et al reported that pre-operative conization could lower disease recurrence by reducing the size of the primary cervical mass. In addition, this procedure could also reduce the potential for tumor cell spillage at the time of uterine manipulation and colpotomy.^[15]^ In the present study, 20 patients (80.0%) in the MIRH group had stage IB1 disease and of these 9 (45.0%) underwent conization, while 2 of the 5 (40.0%) stage IB2-IIA2 cases underwent conization. In contrast, only 25% of patients with stage IB1 disease in the ORH group underwent conization and even less (12.5%) of the stage IB2-IIA patients. The second possible reason for the similar survival outcomes observed for ORH and MIRH patients is that all cervical cancer cases at our institute are routinely evaluated by abdominal CT scan, pelvis MRI, and PET-CT before RH. This could improve the case selection suitable for MIRH. Thirdly, patients in the ORH group may have higher stage disease and additional risk factors. Although not reaching significance, the incidence of stage IB1 disease was slightly higher in the MIRH group (80.0%) than the ORH group (73.6%), while stage IIA1 disease was higher in the ORH group (9.9%) than MIRH (0%, *P* = .309). The incidence of lymph node invasion was significantly higher in the ORH group (37.4%) than in the MIRH group (12.0%, *P* = .016). The resection margin involvement was also more frequent in the ORH group (13.2%) compared to MIRH (0%), resulting in significantly more ORH patients receiving adjuvant therapy after surgery than MIRH patients (71.4% vs 56.0%, respectively, *P* = .002). Fourth, all MIRH procedures carried out during the study period in our institute were performed using vaginal colpotomy. A recent LACC trials reported worse survival for the MIRH group,^[13]^ with intracorporeal colpotomy identified as one of the weak points of MIRH. Kong et al reported a higher rate of disease recurrence in the intracorporeal colpotomy group compared to the vaginal colpotomy group (16% vs 5%, respectively). Of the patients in the intracorporeal group with recurrence, 62% had intraperitoneal spread or carcinomatosis.^[13]^ Other studies have also shown non-inferior PFS and OS by performing vaginal colpotomy during MIRH.^[15]^ Finally, the present study may have a bias due to the relatively small number of patients with stage IB2 - IIA2 disease (i.e., 24 cases in the ORH group (26.4%) and 5 in the MIRH group (20.0%)).

The present study could also have other biases that influenced the results. Despite higher rates of preoperative conization, the possibility of case selection, and a lower incidence of risk factors, MIRH did not show superior oncological outcomes compared to ORH. A possible reason for this was the potential for tumor cell spillage at the time of uterine manipulation and colpotomy. Although not reaching statistical significance, the rate of vaginal stump and pelvic cavity recurrence was higher in MIRH group. Hence, vaginal colpotomy may not be completely effective in preventing tumor spillage.

One of the strengths of this study is that it included all eligible patients who underwent RH, at a single institute since its opening. Second, gynecologic oncologists at this institute have used uterine manipulation and CO_2_ insufflation for MIRH, while vaginal colpotomy and cuff closure have also been used. LARVH (by surgeons SJL and TJK) and LRH (by SHS and KAS) were performed using vaginal colpotomy and cuff closure. The reason for using vaginal colpotomy with MIRH was not because of the potential harm from intracorporeal colpotomy, but due to the skill of the surgeons with this procedure acquired during their training. Prior to the LACC trial, the oncological safety of MIRH was not in doubt.

The current study has several limitations. First, this was a retrospective study with a small number of patients. The initial case selection was determined by the surgeons without apparent selection criteria. Although there were statistically significant similarities in certain patient characteristics that could affect survival, the lack of selection criteria may have introduced some bias in the study. Second, although all surgeons were certified as specialists by the “Korean Society of Gynecologic Oncology,” the surgical methods and surgeon skills were not standardized. The gynecologic oncologists at our institute were all trained in different cancer centers. Third, the relatively small number of disease-related deaths during the follow-up period limits the statistical power of the study. During the study period, only 4 patients died of disease recurrence, with 3 in the ORH group and one in the MIRH group.

In conclusion, MIRH using vaginal colpotomy shows a similar level of oncological safety as ORH in patients with stage IB1-IIA2 cervical cancer. However, although not reaching statistical significance, MIRH showed a trend for worse PFS compared to ORH in stage IB2-IIA2 cervical cancer cases. Furthermore, despite a higher incidence of unfavorable prognostic factors in the ORH group, MIRH did not show superior oncological outcomes. Therefore, it should be borne in mind that MIRH may not be safe in cervical cancer cases, despite the use of vaginal colpotomy. Because of limitations with the study design and the low number of events, future studies will be necessary to draw firmer conclusions.

## Acknowledgments

We thank all the peer reviewers and editors for their opinions and suggestions.

## Author contributions

**Conceptualization:** Sun Joo Lee, Seung-Hyuk Shim.

**Data curation:** Eun Jung Yang, Nae Ry Kim, A Jin Lee.

**Formal analysis:** Eun Jung Yang, Sun Joo Lee, Nae Ry Kim, A Jin Lee.

**Investigation:** Eun Jung Yang, A Jin Lee, Seung-Hyuk Shim.

**Methodology:** Eun Jung Yang, Seung-Hyuk Shim.

**Resources:** Seung-Hyuk Shim.

**Software:** Eun Jung Yang, Nae Ry Kim, Seung-Hyuk Shim.

**Supervision:** Sun Joo Lee.

**Writing – original draft:** Eun Jung Yang.

**Writing – review & editing:** Sun Joo Lee.
